# Contribution of Puma to Inflammatory Resolution During Early Pneumococcal Pneumonia

**DOI:** 10.3389/fcimb.2022.886901

**Published:** 2022-05-26

**Authors:** Daniel E. Kennedy II, Perceus Mody, Jean-Francois Gout, Wei Tan, Keun Seok Seo, Alicia K. Olivier, Jason W. Rosch, Justin A. Thornton

**Affiliations:** ^1^Department of Biological Sciences, Mississippi State University, Starkville, MS, United States; ^2^Department of Comparative Biomedical Sciences, College of Veterinary Medicine, Mississippi State University, Starkville, MS, United States; ^3^Department of Population and Pathobiology, College of Veterinary Medicine, Mississippi State University, Starkville, MS, United States; ^4^Department of Infectious Diseases, St. Jude Children’s Research Hospital, Memphis, TN, United States

**Keywords:** PUMA (p53 upregulated modulator of apoptosis), inflammation, streptococcus pneumoniae (pneumococcus), apoptosis, innate immunity

## Abstract

Apoptosis of cells at the site of infection is a requirement for shutdown of inflammatory signaling, avoiding tissue damage, and preventing progression of sepsis. *Puma^+/+^* and *Puma^-/-^* mice were challenged with TIGR4 strain pneumococcus and cytokines were quantitated from lungs and blood using a magnetic bead panel analysis. *Puma^-/-^* mice exhibited higher lung and blood cytokine levels of several major inflammatory cytokines, including IL-6, G-CSF, RANTES, IL-12, IFN-ϒ, and IP-10. *Puma^-/-^* mice were more susceptible to bacterial dissemination and exhibited more weight loss than their wild-type counterparts. RNA sequencing analysis of whole pulmonary tissue revealed Puma-dependent regulation of *Nrxn2*, *Adam19*, and *Eln*. Enrichment of gene ontology groups differentially expressed in *Puma^-/-^* tissues were strongly correlated to IFN-β and -ϒ signaling. Here, we demonstrate for the first time the role of Puma in prohibition of the cytokine storm during bacterial pneumonia. These findings further suggest a role for targeting immunomodulation of IFN signaling during pulmonary inflammation. Additionally, our findings suggest previously undemonstrated roles for genes encoding regulatory and binding proteins during the early phase of the innate immune response of pneumococcal pneumonia.

## Introduction

During pneumococcal infection, interaction of bacteria with the host epithelium initiates recruitment of neutrophils (Yamamoto et al., 2014). Following recruitment, activated neutrophils are capable of inducing programmed cell death in epithelial cells (Jia et al., 2014). Pneumococcus can induce both direct and indirect apoptosis in neutrophils (Zysk et al., 2000; Engelich et al., 2001), epithelial cells (Schmeck et al., 2004), and macrophages (Marriott et al., 2004), observations that have been mechanistically tied to PLY, cell wall components, and H_2_O_2_ (Marriott and Dockrell, 2006; Rai et al., 2015). In murine pneumococcal pneumonia, macrophage apoptosis has been shown to be crucial for resolution of pulmonary inflammation (Marriott et al., 2006). Induction of apoptosis, depending on cell type, can have detrimental or beneficial results, but coordinated and organized cell death is essential during infection resolution. Apoptosis modulates innate immune signaling, cell fate of responding phagocytes, promotes resolution of inflammation, and limits tissue damage (Haslett, 1999; Kennedy and Deleo, 2009; Esmann et al., 2010; Fox et al., 2010; Bordon et al., 2013; Greenlee-Wacker, 2016). The resultant effects of pneumococcal injury and apoptosis within the tissues strongly correlates with wound healing and repair (Matute-bello and Martin, 2003; Geiser et al., 2004; Wu and Chen, 2014; Domon et al., 2016; Holzmann et al., 2016). Numerous experimental *in vitro*, *in vivo*, and clinical studies have reported several pro-inflammatory cytokines up-regulated during the early phase of pneumococcal pneumonia, including IL-6, G-CSF, TNF-α, IL-1β, IL-12, IFN-ϒ, and IP-10 (Van Der Poll et al., 1997; Ethuin et al., 2004; Yamamoto et al., 2004; Palaniappan et al., 2006; Sun et al., 2007; Endeman et al., 2011; Yamada et al., 2011; Gomez et al., 2015; Skovbjerg et al., 2017; Loughran et al., 2018). However, in the last two decades, over-expression of IL-12, IFN-ϒ, and IP-10 has emerged as a highly potent pro-inflammatory, neutrophil-mediated signaling axis in murine models of pneumococcal pneumonia (Yamamoto et al., 2004; Sun et al., 2007; Seyoum and Yano, 2011; Yamada et al., 2011; Gomez et al., 2015).

Neutrophils are the primary effector cells of the host innate response to pneumococcal pneumonia (Craig et al., 2009). In pneumococcal pneumonia patients, the activation of neutrophils after extravasation into the lungs delays the rate of spontaneous apoptosis (Droemann et al., 2000). However, apoptosis of neutrophils is an essential feature of pneumococcal infection, as apoptotic neutrophils must be cleared from the site upon performing their effector functions (Koedel et al., 2009). Pneumococcus can induce apoptosis in neutrophils in an H_2_O_2_-dependent fashion and accelerate apoptosis by PLY membrane perforation at relatively lower multiplicities of infection (MOI) (Zysk et al., 2000).

PUMA (p53-up-regulated modulator of apoptosis), a pro-apoptotic member of the BH3-only family of the BCL-2 superfamily of eukaryotic proteins, is one of the primary effector proteins that promotes apoptosis (Nakano and Vousden, 2001; Vousden, 2005). In a murine model of pneumococcal infection, neutrophil-mediated protection against lethal dissemination of pneumococci was shown to be Puma-dependent apoptosis (Garrison et al., 2010). However, it is unclear which steps of apoptosis signaling pathways are involved in the early phase interactions between neutrophils and pneumococcus. Subsequently, the effects of Puma-dependent apoptosis on global gene expression and innate immune signaling in the lung during the early phase of pneumococcal pneumonia have not yet been investigated.

In this study, we attempted to characterize Puma-dependent responses in the lung during the early phase of pneumococcal pneumonia. Given the clinical relevance of targeting the cytokine response during the early phase of the innate immune response during acute community acquired pneumonia (Woods and José, 2017), we sought to quantitate differences in inflammatory cytokines in the lungs of *Puma^+/+^* and *Puma^-/-^* mice. Finally, to further understand what Puma-dependent transcriptional responses may be induced during early pneumococcal infection, we attempted to characterize changes in the lung transcriptome.

## Materials and Methods

### Bacterial Culture

*S. pneumoniae* strain TIGR4 (Tettelin et al., 2001) was cultured overnight on tryptic soy agar (TSA) plates supplemented with 5% sheep’s blood (EMD Millipore) and subsequently inoculated into 10 mL pre-warmed C+Y medium (Lacks and Hotchkiss, 1960). TIGR4 was cultured to mid-log, centrifuged for 10 minutes at 15,871 x g and 4°C, and resuspended in 1 mL of C+Y with 20% glycerol (Thermo Fisher Scientific). Resuspended TIGR4 was aliquoted by volumes of 100 μL and stored at -80°C until used in animal challenges. Before challenging animals, one aliquot TIGR4 was thawed on ice, serially diluted in 1X PBS (Thermo Fisher Scientific), cultured on blood agar overnight, and quantitated.

### Statement of Ethics, Animal Handling, and Challenges

All experiments involving animals were planned and conducted in accordance with guidelines of the MSU Institutional Animal Care and Use Committee (Protocol number 19-537). The committee-approved procedures performed in the study presented here. Puma heterozygote mice were crossed and resulting C57BL/6 or *Puma^-/-^* (The Jackson Laboratory, C57BL/6-Bbc3^tm1Ast^/J, RRID: IMSR_JAX: 011067) mice from generations F5 and F6 were used in challenges. Animals were maintained at Biosafety Level 1 and 2 facilities in the Mississippi State University Animal Facility at Harned Hall. Animals were housed among litter mates until isolated overnight for challenge. On the days of challenges, TIGR4 aliquots were removed from -80°C, thawed on ice, and resuspended in 900 μL 1X PBS, then further diluted to a working dilution of 2 x 10^6^ CFU/mL. Fifty microliters of working dilution TIGR4 or 1X PBS were administered intranasally. Twenty-four hours post-infection (p.i.), signs of disease and body weight were recorded immediately before euthanasia. To determine CFU within tissues, nasopharyngeal tissues were aseptically flushed with filter-sterilized 1X PBS and collected in-tube, pulmonary tissues mechanically homogenized in 1 mL 1X PBS using an electric tissue homogenizer, and blood was drawn retroorbitally. Crude samples (bood or homogenized respiratory tissues) were appropriately diluted to determine CFU titres onto TSA supplemented with 5% sheep’s blood and incubated overnight at 37°C under CO_2_. Tracheae were surgically transversely severed and whole pulmonary tissues were prepared as follows: transferred to 1X PBS to be homogenized for cytokine analysis; perfused with and homogenized in Trizol (Thermo Fisher Scientific) reagent for RNA extraction and library preparation; perfused with and transferred to 10% neutral buffered formalin (Thermo Fisher Scientific) for paraffin embedding.

### Histopathological Analysis

Tissues were collected, fixed in 10% neutral buffered formalin, routinely processed, embedded in paraffin, sectioned at 5 μm and stained with hematoxylin and eosin (Cardiff et al., 2014). Lung sections were scored for inflammation based on the following parameters: patchy interstitial hypercellularity due to increased mononuclear cells (0-absent; 1-present), perivascular suppurative inflammation with edema (0-absent; 1-present but focal (3 or fewer vessels affected); 2-present – extensive (more than 3 vessels affected), vasculitis (0-absent; 1-present) and increased alveolar macrophages (0-absent; 1-present). All scoring was done blinded to genotype (**Supplementary Table S1**).

### Cytokine Analysis

To characterize the pneumococcal-induced cytokine profile 24 hours p.i., pulmonary tissues were harvested as described above and blood was retroorbitally drawn. Crude pulmonary tissue homogenates prepared as described above were centrifuged for 10 minutes at 9,400 x g and 4°C. Supernatants were transferred to clean tubes and stored at -80°C for analysis. Tissue homogenate pellets were washed once with 1X PBS and centrifuged for 10 minutes at 21,130 x g and 4°C. Whole tissue lysates were centrifuged for 4 minutes at 690 x g. Whole tissue lysates were transferred to clean tubes and stored at -80°C for analysis. Blood samples were centrifuged for 3 minutes at 1,550 x g. Sera were transferred to clean tubes and store at -80°C for analysis. Concentrations of granulocyte-colony stimulating factor (G-CSF), granulocyte macrophage-colony stimulating factor (GM-CSF), interleukin-1 alpha (IL-1α), IL-1β, IL-2, IL-4, IL-5, IL-6, IL-9, IL-10, IL-12p40, IL-12p70, IL-13, IL-15, IL-17, interferon-inducible protein-10 (IP-10), keratinocyte-derived chemokine (KC), monocyte chemotactic protein-1 alpha (MCP-1α), macrophage inflammatory protein-1alpha (MIP-1α), MIP-1β, MIP-2, regulated on activation normal T cells expressed and secreted (RANTES), tumor necrosis factor-alpha (TNF-α), and interferon-gamma (IFN-ϒ) were determined using the MILLIPLEX MAP Mouse Cytokine/Chemokine Magnetic Bead Panel (EMD Millipore # MCYTOMAG-70K) according to the manufacturer’s instructions.

### RNA Isolation and Library Preparation

To characterize the post-pharyngeal pulmonary transcriptome 24 hour p.i., tissues were mechanically homogenized using an electrical tissue homogenizer in 5 mL Trizol reagent. From crude pulmonary homogenates, 1mL was transferred to a clean tube and incubated at room temperature for 5 minutes. The remaining crude Trizol homogenate was stored at -80°C for curation and further analysis. Two-hundred microliters chloroform (Thermo Fisher Scientific) were added, shaken, and incubated at room temperature for 2 minutes. Samples were centrifuged 20 minutes at 15,900 x g and 4°C. RNA was purified using the RNeasy kit (Qiagen) according to the vendor’s instructions. RNA purity was determined using a Qubit 2.0 fluorometer and Qubit RNA BR and dsDNA HS assay kits (Thermo Fisher Scientific). Ten micrograms total RNA served as starting material with the NEBNext Poly(A) mRNA Magnetic Isolation Module (New England Biolabs), and cDNA libraries were generated using the NEBNext Ultra RNA Library Prep Kit for Illumina (New England Biolabs). Samples of cDNA from all mice were normalized for concentration, pooled, and sequenced on their own lane of a NOVASeq 6000 sequencer at 100 base-pair paired-end reads and yielded at least 10 million reads.

### Demultiplexing, Alignment, Normalization, & Bioinformatics Analysis

RNAseq reads were trimmed with BBDuk (part of BBMap version 38.5) for both quality (minimum quality set to 20 with: “qtrim=rl trimq=20”) and adapters trimming. Transcript abundances for each sample were computed with Kallisto version 0.45.0 using default parameters (kallisto quant –bias) for pseudoalignment against the *Mus musculus* annotated transcripts from the Ensembl database (GRCm39, release 103) (Bray et al., 2016; Yates et al., 2020). Differentially expressed genes were detected using the DESeq2 package (Love et al., 2014). Clustering of samples based on gene expression patterns was performed with the vsn and pheatmap packages and revealed that 3 samples of infected mice clustered with mock-infected mice. Blood CFU data confirmed that the infection status of these three mice was questionable, and they were removed from the analysis (**Table 1**). Gene Ontology enrichment was performed using the topGO package in R, using the ks/classic statistic/algorithm combination.

**Table 1:** CFU counts per individual mice used in RNAseq pneumococcal challenges.

Mouse_id	Stimulus	i7_primer	i7_index	i5_primer	i5_index	CFU_blood
*854WTM*	Spn	i701	ATTACTCG	i501	TATAGCCT	8.00E+03
*859WTF*	Spn	i703	CGCTCATT	i501	TATAGCCT	5.10E+04
*860WTM*	Spn	i702	TCCGGAGA	i501	TATAGCCT	2.34E+05
*864WTF*	Spn	i704	GAGATTCC	i501	TATAGCCT	0.00E+00
*868WTF*	Spn	i705	ATTCAGAA	i501	TATAGCCT	6.00E+03
*406KOM*	Spn	i703	CGCTCATT	i501	TATAGCCT	2.46E+05
*413KOF*	Spn	i704	GAGATTCC	i501	TATAGCCT	0.00E+00
*867KOF*	Spn	i701	ATTACTCG	i503	CCTATCCT	1.24E+05
*870KOF*	Spn	i705	ATTCAGAA	i501	TATAGCCT	0.00E+00
*872KOM*	Spn	i702	TCCGGAGA	i501	TATAGCCT	1.65E+05
*408WTM*	PBS	i703	CGCTCATT	i505	AGGCGAAG	NA
*415WTF*	PBS	i709	CGGCTATG	i502	ATAGAGGC	NA
*889WTF*	PBS	i708	TAATGCGC	i502	ATAGAGGC	NA
*890WTM*	PBS	i702	TCCGGAGA	i504	GGCTCTGA	NA
*894WTM*	PBS	i707	CTGAAGCT	i502	ATAGAGGC	NA
*402KOF*	PBS	i706	GAATTCGT	i502	ATAGAGGC	NA
*409KOF*	PBS	i709	CGGCTATG	i502	ATAGAGGC	NA
*411KOM*	PBS	i710	TCCGCGAA	i502	ATAGAGGC	NA
*893KOM*	PBS	i708	TAATGCGC	i502	ATAGAGGC	NA
*900KOF*	PBS	i707	CTGAAGCT	i502	ATAGAGGC	NA

WT and KO mice were challenged with 2 x 10^5^ total CFU TIGR4 or mock-infected with equivalent volumes of PBS. Lungs were harvested as described in methods 24 h p.i. CFU were quantitated from blood. Outliers are shown in red. For each group, WT (n = 5), KO (n = 5).

NA, Not applicable.

## Results

### PUMA Limits Pneumococcal Inflammation & Sepsis

**Figure 1:**
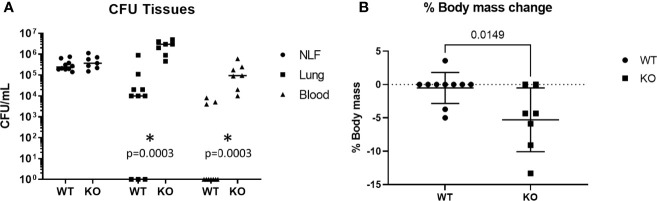
Puma attenuates sepsis and morbidity. **(A)** Mice were intranasally challenged with 2 x 10^5^ total CFU. Pneumonia was allowed to develop for 24 h, at which time mice were euthanized and tissues were sampled. For each group, WT (*n* = 10), KO (*n* = 7). Horizontal lines represent means. A Mann-Whitney U test was performed, α = 0.05. **(B)** Mice were weighed immediately before challenge and before sacrifice. A two-tailed, unpaired T-test was performed, α = 0.05. *p = 0.0003.

To investigate the role of Puma during early pneumococcal infection, *Puma^+/+^* (WT) and *Puma^-/-^* (KO) mice were intranasally infected with 2 x 10^5^ total CFU TIGR4. At 24 hours p.i., TIGR4 CFU were quantitated from nasal lavage fluid (NLF), pulmonary homogenates, and blood. While numbers of TIGR4 recovered from NLF were similar among all mice, *Puma^-/-^* mice demonstrated significantly higher bacterial counts in the lungs and bloodstream at 24 hours p.i. compared to *Puma^+/+^* mice (**Figure 1A**). In the same timeframe, only 30% of *Puma^+/+^* mice exhibited TIGR4 disseminated to blood, compared to 100% of *Puma^-/-^* mice. *Puma^-/-^* mice lost significantly more weight (mean of 1.42 g) than *Puma^+/+^* mice (mean of 0.2 g) over the 24-hour course of infection (**Figure 1B**) indicating excessive disease burden. These data suggest that pneumococcus is more invasive in *Puma^-/-^* mice.

**Figure 2:**
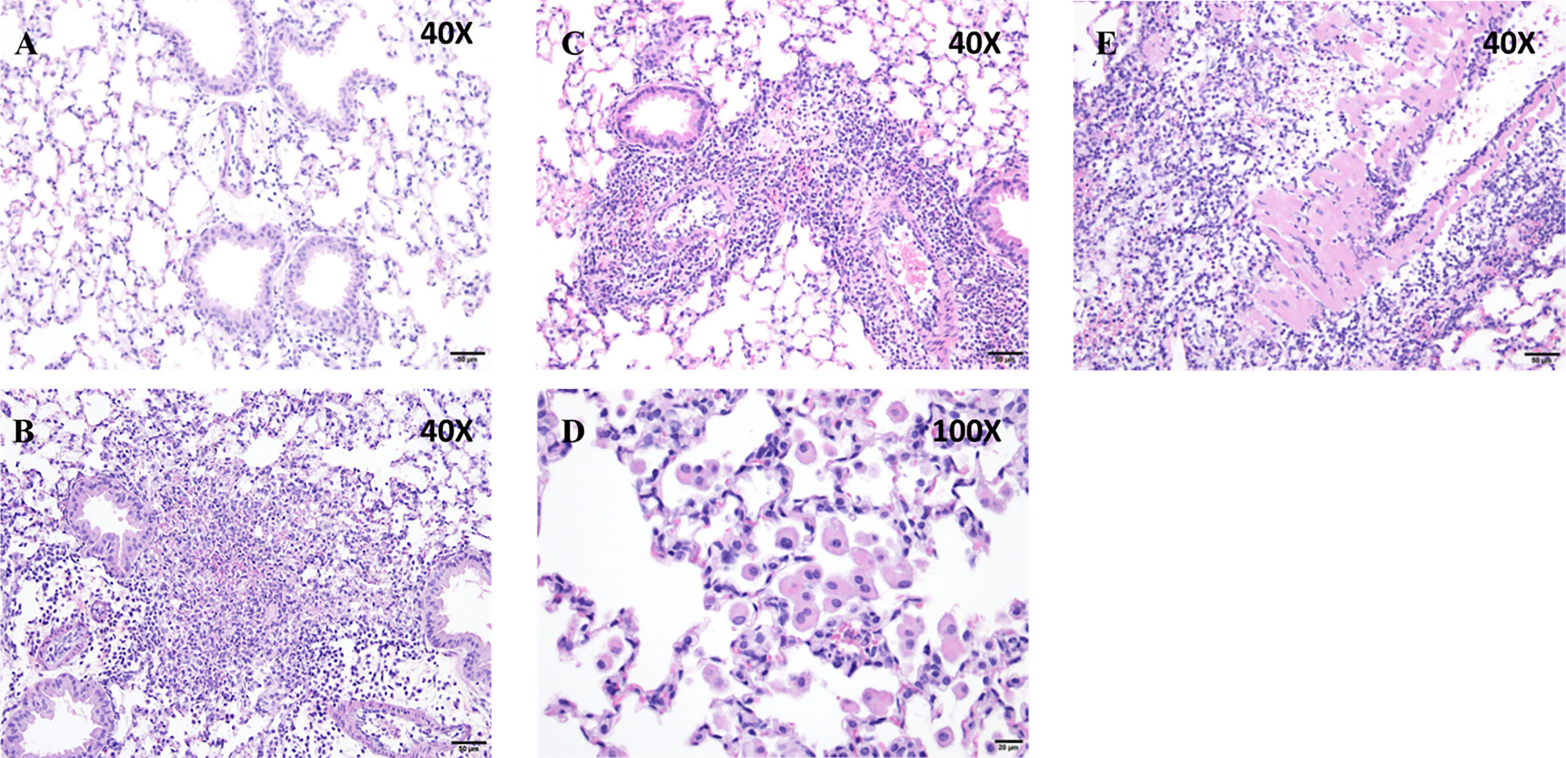
Puma protects against tissue inflammation. **(A, B)** *Puma^+/+^* mice exhibited mild peribronchioloar inflammation with edema and neutrophils and macrophages. **(C–E)** *Puma^-/-^* mice exhibited moderate peribronchiolar/perivascular inflammation, abundant neutrophils, macrophages, vasculitis (eosinophilic material, vascular thickening). Scale bars: **A–C**, **E**, 50 μm; **D**, 20 μm. Images shown are representative of the pathology described in the results from 3 sections each from 5 mice.

Histological analysis showed that peribronchiolar and perivascular inflammation was noted in both *Puma^+/+^* and *Puma^-/-^* lungs (**Figure 2**). In the *Puma^+/+^* group, one of five mice had no histologic changes (**Figure 2A**), while four of five had patchy areas of increased cellularity of the interstitium with three of five classified as mild (**Figure 2B**). No evidence of vasculitis was detected in *Puma^+/+^* lungs. In the *Puma^-/-^* group, one of five mice had increased alveolar macrophages as the only histologic change (**Figure 2D**), while four of five had patchy areas of increased cellularity of the interstitium with two of five lungs scored moderate (**Figure 2C**). One *Puma^-/-^* mouse exhibited evidence of vasculitis (**Figure 2E**).

### Puma Dampens Cytokine Signaling and Prevents Onset of the Cytokine Storm

**Figure 3:**
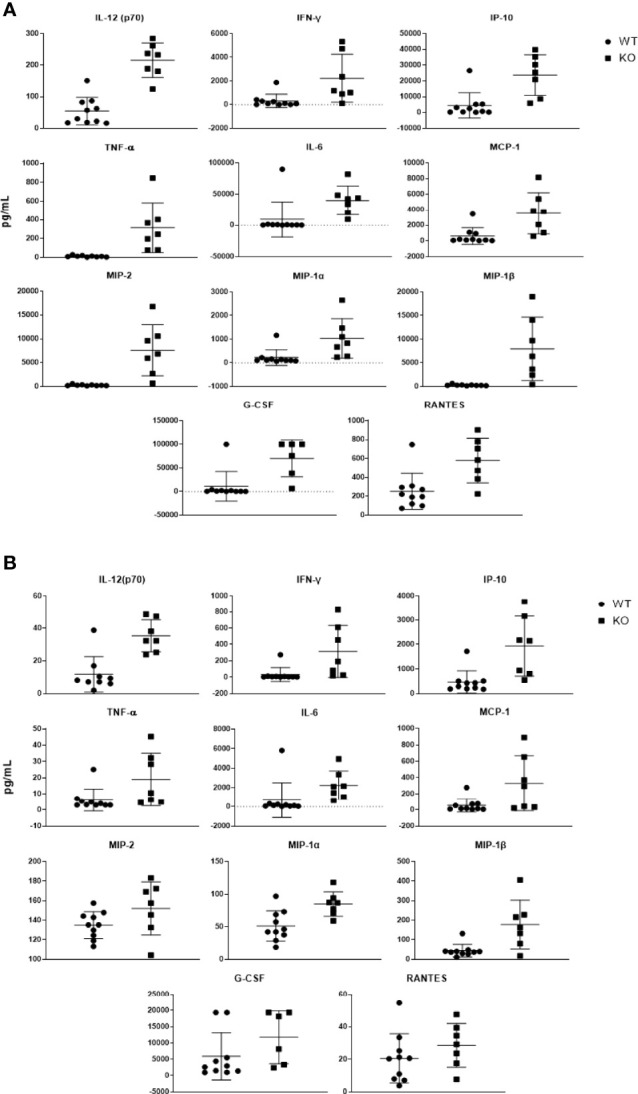
Puma-dependent host responses during early pneumococcal pneumonia suppress production of cytokines in the lung. Cytokines expressed more than 3-fold in *Puma^-/-^* mouse lungs **(A)** and sera **(B)** are shown here. Cytokines were detected *via* magnetic bead panel using flow cytometry analysis. Values are reported as pg/mL. Mann-Whitney U tests were performed to determine significance, α = 0.05.

To characterize the effect of Puma on the cytokine response elicited by pneumococcus, lung and blood cytokine levels from *Puma^+/+^* and *Puma^-/-^* mice were quantitated 24 hours p.i. (**Table 2**). The cytokines IL-1β, -6, -12, -13, IP-10, MCP-1, MIP-1α, -1β, TNF-α, and IFN-ϒ were significantly over-expressed in *Puma^-/-^* lungs and blood compared to those of *Puma^+/+^*. Similarly, in *Puma^-/-^*, differences in G-CSF, GM-CSF, IL-1α, -17, KC, MIP-2, and RANTES were significant only in lung, while IL-9, -10, and -12(p40) were only significantly different in blood. Differences in IL-2, -4, -5, and -15 were observed in *Puma^+/+^* and *Puma^-/-^* mouse lungs and blood, all concentrations being higher in *Puma^-/-^* tissues, but none were statistically significant (**Figure 3**).

**Table 2:** Pro-inflammatory cytokine profile of *Puma^-/-^* mice during early innate immune response.

Cytokine	Higher in *Puma^-/-^* (*)		Known or suspected role in innate response during pneumococcal pneumonia
	*Blood*	*Lungs*	
G-CSF		*	Activates PMN and reduces pro-inflammatory cytokines IL-1β, TNF-α, KC (Knapp et al., 2004)
GM-CSF		*	Enhances PMN recruitment, decreases Spn burden, protects pulmonary macs and PMN from Spn-induced apoptosis (Steinwede et al., 2011)
IL-1α		*	Induced by PLY, enhances clearance of colonization by activating IL-1 signaling (Kuipers et al., 2018)
IL-1β	*	*	Enhances clearance of colonization and Th17-induced recruitment of macs (Lemon et al., 2015)
IL-2			Unknown
IL-4			Unknown
IL-5			Unknown
IL-6	*	*	Promotes inflammation during early infection (Wang et al., 2005)
IL-9	*		Unknown
IL-10	*		Reduces infiltration of activated PMN and production of pro-inflammatory cytokines (Penoloza et al., 2015; Penoloza et al., 2018)
IL-12 (p40)	*		Essential for functional IL-12 (p70) PMN-dependent recruitment (Yamamoto et al., 2004)
IL-12 (p70)	*	*	Enhances PMN recruitment and stimulates IFN-ϒ production (Sun et al., 2007; Loughran et al., 2018)
IL-13	*	*	Unknown
IL-15			Inhibits Spn-induced apoptosis in lung epithelial cells (Hocke et al., 2008)
IL-17		*	Induced by PLY (Dockrell et al., 2012); Augments neutrophil recruitment (Ritchie et al., 2018)
IP-10	*	*	Spn strain-dependent bactericidal activity (Bruce et al., 2018); Induced in PMN (Gomez et al., 2017)
KC		*	Induced by Spn H_2_O_2_ in epithelial cells (Loose et al., 2014)
MCP-1	*	*	Recruits MDM to alveoli, reduces Spn burden, and improves survival (Winter et al., 2007)
MIP-1α	*	*	Increases MDM,PMN recruitment (Williams et al., 2015)
MIP-1β	*	*	Increases MDM,PMN recruitment (Williams et al., 2015)
MIP-2		*	Increases PMN recruitment (Dallaire et al., 2001)
RANTES		*	Augments colonization clearance (Pallaniappan et al., 2006); Recruits PMN (Koppe et al., 2012)
TNF-α	*	*	Reduces Spn burden and enhances PMN recruitment (Jeong et al., 2015)
IFN-ϒ	*	*	Augments PMN-mediated clearance (Yamada et al., 2010)

Lungs and blood were harvested from mice challenged with TIGR4 pneumococcus 24 p.i. More than 80% of cytokines probed were significantly higher in Puma^-/-^ tissues. α = 0.05.

### Loss of Puma Induces Changes in the Pulmonary Transcriptome

**Figure 4:**
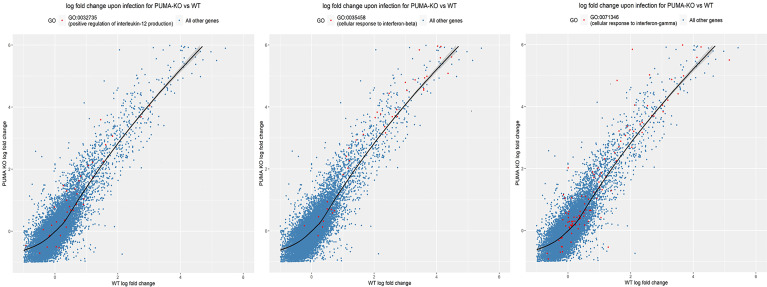
Gene ontology analysis reveals Puma limits IL-12 and Type I and II IFN signaling. Genes significantly expressed (red dots) in their respective GO groups are plotted against all other genes (blue dots). Significance was determined using the Kolmogorov-Smirnov test and p-values were adjusted for multiple-testing. The area above the black line indicates higher expression in *Puma^-/-^* mice, while the area under the black line indicates higher expression in *Puma^+/+^* mice.

To further investigate Puma-dependent transcriptional responses during the early phase of pneumonia, total RNA was extracted from lungs of five mice per challenge group 24 hours p.i., purified, and pooled for RNA-seq analysis. Using DEseq2, 21 differentially expressed genes, 15 of which had annotations, were detected with an adjusted p-value < 0.05 (**Table 3**). Of note, the genes *Nrxn2*, *Adam19*, and *Eln* were each up-regulated in *Puma^+/+^* lungs and down-regulated in those of *Puma^-/-^* mice. We performed a gene ontology (GO) analysis on all three sub-ontologies: Biological Process (BP), Molecular Function (MF), and Cellular Component (CC). We found 28, 4, and 7 GO categories significantly enriched for genes differentially regulated in *Puma^-/-^* mice in BP, MF, and CC, respectively (**Supplementary Table S2**). To focus on the categories most likely to have a biologically significant contribution to the infection response, we added a fold-enrichment (FE) criteria and retained only GO sub-categories with FE > 2.5. This resulted in 16, 4, and 3 categories being retained, respectively, in the BP, MF, and CC sub-ontologies. In accordance with detection of IL-12 cytokine, the GO group ‘positive regulation of IL-12 production’ (GO:0032735) was enriched 3.6 fold (**Figure 4**). Notably, GO groups ‘cellular response to IFN-β’ (GO:0035458) and ‘cellular response to IFN-ϒ’ (GO:0071346) were enriched 5.46 and 3.45, respectively. Individual lists of gene expression from each of these GO groups are shown in **Supplementary Table S3**.

**Table 3:** Differentially expressed genes in *Puma^-/-^* lungs.

Gene	baseMean	log2FoldChange	lfcSE	stat	pvalue	padj
Nrxn2 *	278.41809	-1.6401589	0.2631083	-6.233778	0.00E+00	0.0000078
***Prrt2***	60.30429	-2.4003748	0.4447364	-5.397298	1.00E-07	0.0005823
Adam19 *	1378.17251	-0.6234511	0.1175516	-5.303639	1.00E-07	0.0006413
Eln *	3187.73317	-1.6045925	0.3054167	-5.253781	1.00E-07	0.0006413
Grk4 *	142.06737	-1.6525749	0.3551424	-4.653274	3.30E-06	0.0070303
***Olfr2***	23.35589	2.4365302	0.5408849	4.504711	6.60E-06	0.0115342
***Mturn***	981.39638	-0.8387504	0.1914976	-4.379953	1.19E-05	0.0185773
***Cracr2b***	1193.88323	-0.9459835	0.220296	-4.294147	1.75E-05	0.0232225
***Tmem204***	2313.99531	-1.1646703	0.2736711	-4.25573	2.08E-05	0.0256219
***Aoah***	155.09042	1.8136874	0.4283164	4.234457	2.29E-05	0.0262935
***Rab8b***	1760.31877	0.8942729	0.2158181	4.143641	3.42E-05	0.0338506
***Nckap5l***	225.00742	-1.4002127	0.336441	-4.161837	3.16E-05	0.0338506
***Triobp***	865.65297	0.926385	0.2250176	4.116945	3.84E-05	0.0347858
***Tbc1d1***	896.23624	0.9062004	0.2232808	4.058569	4.94E-05	0.0424989
***Crybg1***	829.49315	1.0914384	0.2717405	4.016472	5.91E-05	0.0484281

Lungs and blood were harvested from mice challenged with TIGR4 pneumococcus 24 p.i. Gene symbols with asterisks indicate transcripts up-regulated in Puma^+/+^ and down-regulated in Puma^-/-^. α = 0.05.

## Discussion/Conclusion

Apoptosis is integral in the host defense response against pneumococcal infection. Neutrophils are the primary cell type responsible for direct clearance of pneumococcus from the lung (Garrison et al., 2010). Phagocytosis of pneumococcus induces apoptosis in neutrophils *via* phagocytosis-induced cell death (PICD) (Kobayashi et al., 2017). As neutrophils become apoptotic, they are rapidly cleared by macrophages to avoid the detrimental effects of secondary necrosis and prolonged inflammation within the lung (Savill, 1997; Henson and Bratton, 2013). Neutrophil apoptosis directly and indirectly signals surrounding cells to aid in resolution (Henson and Bratton, 2013; Jia et al., 2014). Apoptosis of other cell types also contributes to pneumococcal infection resolution, including macrophages (Marriott et al., 2004; Rai et al., 2015), epithelial cells (Schmeck et al., 2004; Marriott and Dockrell, 2006), and T-cells (Kemp et al., 2002). During the early innate immune response in the lung, all of these cell types may contribute to apoptosis-induced signaling. Multiple cell types have been documented undergoing PUMA-induced apoptosis, including epithelial cells (Dang et al., 2020), neutrophils (Garrison et al., 2010), and T-cells (Fischer et al., 2008).

PUMA distinctly contributes to protective infectious disease responses during acute viral infection by herpes simplex virus (HSV-1) in T-cells (Fischer et al., 2008), and degradation of PUMA induced by *Chlamydia trachomatis* contributes to persistent infection (Dong et al., 2005). However, PUMA-induced apoptosis contributes to *Helicobacter pylori* pathogenesis during gastritis (Dang et al., 2020). These opposing roles of PUMA during infection may lie in the pathophysiology of the respective pathogen and the cell types involved. Puma-deficient mice have been intensively studied for their resistance to cancer-causing stimuli (Garrison et al., 2010). HSV-1 can induce oral cancer in mice and is a suspected causative cancer agent in humans (Fischer et al., 2008), and *H. pylori* is now recognized as a causative agent of gastric cancer. *C. trachomatis* is an obligate intracellular pathogen and epidemiologically correlated with cancers of the female urogenital epithelium, and almost exclusively infects epithelial cells (Zhu et al., 2016). HSV-1 can invade most cell types but primarily infects epithelial cells and resides in neurons as long-term reservoirs within the host. *H. pylori* and pneumococcus are both generally acknowledged as extracellular pathogens and each induces strong neutrophil recruitment, which exacerbates local tissue damage. The up- and down-stream inducers of *puma* in the context of the immune response to infection are currently poorly understood. The contradictory observations on the *Puma^-/-^* mouse model in cancer studies versus our pneumococcal pneumonia model continue to make the Puma phenotype and its up- and down-stream regulators molecules of interest. Our findings support the notion that Puma’s role in infectious disease immunobiology is multifactorial and further demonstrates that loss of Puma may enhance risk of negative health outcomes during infection. The precise pathways involved in the protective phenotype of Puma remain to be thoroughly investigated in the context of infection.

While many cytokines have been associated with inflammation modulation during pneumococcal infection, the pro-inflammatory IL-12/IFN-ϒ/IP-10 signaling axis has emerged as a potent arm of the innate immune response. Infection-induced IL-12 secretion was first identified from dendritic cells and neutrophils and can be induced by pneumococcus *via* TLR3 activation (Rijneveld et al., 2002; Groom and Luster, 2011). The effects of increased IL-12 during pneumococcal pneumonia also include stimulating IFN-ϒ-dependent neutrophil recruitment through increased abundance of MIP-2 and TNF-α (Ethuin et al., 2004; Sun et al., 2007). At 24 hours p.i. *Puma^+/+^* mice expressed less IL-12 in their lungs than *Puma^-/-^*. This could be due to the anti-inflammatory effects of apoptotic neutrophils, as pro-inflammatory signaling is dampened once apoptosis is initiated in neutrophils (Haslett, 1999). In accordance with this notion, we previously demonstrated that *Puma^-/-^* lungs contained more neutrophils than their *Puma^+/+^* counterparts (Garrison et al., 2010). However, pro-inflammatory efferocytosis of neutrophils also induces an IL-12^low^ phenotype in macrophages (Filardy et al., 2010). This likely contributes to the low levels of IL-12 in *Puma^+/+^* lungs following infection, but the precise mechanisms of temporal IL-12 production and its effect on cell death in specific cellular subsets within the lung during early pneumococcal pneumonia need to be further investigated.

The importance of IFN-ϒ signaling is well demonstrated during the early host response to pneumococcal infection. A previous study showed that pneumococcal infection induced significant increases of IFN-ϒ from greater than 98% of neutrophil in the lungs at 24 hours p.i (Yamada et al., 2011). Neutrophilic production of IFN-ϒ during early pneumococcal pneumonia is likely mediated through NADPH oxidase activity, suggesting IFN-ϒ production in neutrophils may be ROS-dependent, the generation of which is also a result of induction of apoptosis (Gomez et al., 2015). Production of IFN-ϒ in pneumococcal pneumonia models has been linked to strain-dependent effects, and suppresses pneumococcal outgrowth during the early phase by recruiting neutrophils (Rubins and Pomeroy, 1997; Groom and Luster, 2011). As a ligand for the CXCR3 receptor, IP-10 can recruit and activate effector CD4^+^ Th1, CD8^+^ CTL, natural killer (NK), and the hybrid NK-T cells (Christaki et al., 2015). In human neutrophils, the combination of IFN-ϒ and TNF-α is the most potent inducer of IP-10 secretion (Cassatella et al., 1997; Gasperini et al., 2019). In accordance with this observation, IFN-ϒ and TNF-α were significantly higher in *Puma^-/-^* mice. IP-10 expression is also strain-dependent and associated with acute lung injury during early pneumococcal pneumonia (Seyoum and Yano, 2011). Interestingly, secretion of IL-12 and IP-10 are increased by human peripheral blood monocytes upon exposure to intact pneumococci, but inhibited when exposed to autolyzed pneumococci (Skovbjerg et al., 2017). In pneumococcal pneumonia, IFN-ϒ signaling is thought to exert beneficial effects by activating NK-T cells but detrimental effects upon activating NK cells (Christaki et al., 2015). However, a role for Puma in these cell types has not yet been characterized. It should be noted, though, that in an experimental pneumonia murine model at 24 hours p.i., T cells, NK cells, and macrophages did not produce IFN-ϒ (Gomez et al., 2015). The increase of IFN-ϒ and subsequent IP-10 production in *Puma^-/-^* mice 24 hours p.i. underscores the importance of apoptosis in resolving inflammation. While both groups displayed increased interstitial inflammation, overall, *Puma^-/-^* mice exhibited a greater degree of inflammation with multiple foci and vasculitis. The excessive inflammation observed in the *Puma^-/-^* mice is likely a result of accumulation of neutrophils that remain active and continue to propagate the IL-12/IFN-ϒ/IP-10 axis. Previous works of others using murine models and data presented here strongly suggest that PUMA-dependent neutrophil apoptosis suppresses the IL-12/IFN-ϒ/IP-10 signaling axis, thereby limiting inflammation and consequent pneumococcal dissemination. However, it is currently unknown if the reported IL-12/IFN-ϒ/IP-10 signaling in mice recapitulates the acute-phase cytokine signaling in humans. Previously, IFN-ϒ had been ruled out as an acute-phase cytokine in humans (Endeman et al., 2011). Although, another clinical study found significant differences in bronchoalveolar lavage fluid IFN-ϒ between healthy control subjects and those with CAP and in serum between severe and non-severe CAP patients upon admission (Paats et al., 2013). Beyond the intrinsic differences in murine and human physiology, a likely explanation for these differences may be the temporal differences in surveillance and detection of infection in humans, with testing only occurring within about 72 hours of the first onset of symptoms (Mandell et al., 2007). Consequently, the mechanistic details of apoptotic suppression of the IL-12/IFN-ϒ/IP-10 pro-inflammatory axis, temporospatial regulation of the intercellular signaling, and whether the cascade results in feedback in humans during development of bacterial pneumonia remain unknown.

Puma-dependent responses also regulate the pulmonary transcriptome. In the lung, ADAM19 is expressed in a variety of cell and tissue types. ADAM19 is expressed higher in bronchiolar than alveolar epithelial cells but expressed higher in smooth muscle cells than in bronchiolar epithelial cells (Dijkstra et al., 2009). However, ADAM19 is only weakly expressed in vascular endothelial cells. The detection of ADAM19 on the apical surfaces of airway epithelial cells may suggest a role in the positive regulation in the early innate immune response to infection since ADAM19 can induce inflammation *via* shedding of TNF-α (Becherer et al., 2003). ADAM19 also contributes to angiogenesis, neurogenesis, synaptogenesis, and adhesion of and invasion by leukocytes (Shirakabe et al., 2001; Dreymueller et al., 2012; Fukazawa et al., 2013). Several of the other ADAM family members have reportedly been observed modulating the immune response to multiple infectious agents, including pneumococcus (Aljohmani and Yildiz, 2020). Down-regulation of *Adam19* in *Puma^-/-^* lungs suggests an immunomodulatory role in the early innate immune response that is short-lived due to yet unidentified Puma-dependent responses to pneumococcal infection. *Nrxn2*, a member of the neurexin gene family, encodes a transmembrane protein on pre-synaptic neurons that forms a Ca^2+^-dependent complex with the protein neuroligin on post-synaptic neurons (Bottos et al., 2009). In addition to regulation of synaptic transmission in the nervous system, neurexin-neuroligin complexation has been observed in vascular tissue. Each neurexin gene encodes a long (α) and short (β) isoform. While the sequence mapping could not discern *Nrxn2* α or β transcripts in the analysis reported here, β isoforms have been detected in complexes with neuroligin in the vascular system while positively modulating angiogenesis in conjunction with vascular endothelial growth factor and increasing vascular tone (Berk et al., 2005; Graziano et al., 2013). *Eln*, the gene encoding elastin, was also up-regulated in a Puma-dependent manner. One explanation for this observation could be that the accumulation of neutrophils in the lung and the hypoxic nature of pneumonia suppress elastin fiber deposition and repair (Berk et al., 2005; Benjamin et al., 2021). Together, these data suggest a Puma-dependent integrated systems physiologic host response to pneumococcal pneumonia. However, the details of these intermolecular and intercellular interactions within the lung have not been thoroughly interrogated during infection.

For the first time, we have demonstrated the immunomodulatory effects of Puma during pneumococcal pneumonia. Puma-dependent responses suppressed pro-inflammatory cytokine signaling, including the highly inflammatory, neutrophil-mediated IL-12/IFN-ϒ/IP-10 signaling axis. Dissemination of pneumococcus and erosion of the pulmonary vasculature was attenuated by Puma-dependent responses. The precise, temporal expression and regulation of IFN-inducible genes remains to be elucidated in the context of Puma-dependent resolution of inflammation. Finally, the activity of ADAM19 during pneumococcal and other bacterial infections also requires further, more intensive study, as well as to what degree, if any, NRXN2 is playing in pulmonary blood pressure regulation during the early phase of acute pneumonia.

## Data Availability Statement

The sequencing raw reads have been deposited in the NCBI Short Read Archive under Bioproject accession number: PRJNA748408.

## Ethics Statement

The animal study was reviewed and approved by Mississippi State University Institutional Animal Care and Use Committee.

## Author Contributions

DK, PM, WT, and JR obtained the data. DK, J-FG, AO, JR, KS, and JT, performed the data analysis. DK, JT, and KS wrote the manuscript. JT was responsible for study concept and design. JT was a supervisor of laboratory analysis of this study. JT is the guarantor of this study and, as such, had full access to all the data in the study and takes responsibility for the integrity of the data and the accuracy of the data analysis. All authors participated in data interpretation and review of the manuscript and approved the final manuscript.

## Funding

Research reported in this publication was supported by the Center for Biomedical Research Excellence (COBRE) in Pathogen–Host Interactions, National Institute of General Medical Sciences, National Institutes of Health (P20 472 GM103646-06). The content is solely the responsibility of the authors and does not necessarily represent the official views of the National Institutes of Health.

## Conflict of Interest

The authors declare that the research was conducted in the absence of any commercial or financial relationships that could be construed as a potential conflict of interest.

## Publisher’s Note

All claims expressed in this article are solely those of the authors and do not necessarily represent those of their affiliated organizations, or those of the publisher, the editors and the reviewers. Any product that may be evaluated in this article, or claim that may be made by its manufacturer, is not guaranteed or endorsed by the publisher.

## Supplementary Material

Supplementary Table 1 Histopathology report from lung sections of WT and KO mice challenged with *S. pneumoniae.* Lung sections were prepared as described in the main text and scored for inflammation. Histology scores from inflamed WT and KO lungs are plotted with mean (horizontal line) and standard deviation (error bars).

Supplementary Table 2 GO enrichment analysis in KO pulmonary tissues. BP, MF, and CC GO categories and their respective significant differentially expressed genes are listed. Adjusted p-values and fold-enrichment scores are shown.

Supplementary Table 3 From **Figure 4** in the main text, individual gene identities represented by red dots plotted for each of the three significantly different GO groups between WT and KO mice are provided. Gene expression differences are shown as log-fold change relative to PBS mock-infected WT or KO cohorts.
